# Effects of Nebulization With Ketamine and Budesonide on Postoperative Sore Throat in Patients Undergoing Elective Surgeries Under General Anesthesia: A Randomized Controlled Comparative Clinical Study

**DOI:** 10.7759/cureus.72195

**Published:** 2024-10-23

**Authors:** Sanya Varma, Subha Teresa Jose Vazhakalayil

**Affiliations:** 1 Anesthesiology, Dr. D. Y. Patil Medical College, Hospital & Research Centre, Dr. D. Y. Patil Vidyapeeth (Deemed to be University), Pune, IND

**Keywords:** budesonide, general anesthesia, ketamine, nebulization, postoperative sore throat

## Abstract

Background

Postoperative sore throat (POST) is a frequent issue after endotracheal intubation, caused by irritation and minor trauma to the throat. Various medications are used to prevent POST by reducing inflammation. This study evaluates the effectiveness of preoperative nebulization with ketamine and budesonide in minimizing POST over time.

Methods

This hospital-based, prospective, double-blinded randomized control trial was conducted with 100 patients aged 18 to 60 years undergoing elective surgeries requiring endotracheal intubation, aiming to compare the effectiveness of nebulization with ketamine and budesonide in reducing POST after oral endotracheal intubation. Patients were randomized into two groups, and POST was assessed using a four-point scale at multiple intervals post-extubation. Hemodynamic parameters were also monitored. Data were analyzed using IBM SPSS Statistics, with significance set at p < 0.05.

Results

In this study, the mean age of the participants was 41.34 ± 12.84 years in one group and 38.84 ± 13.95 years in the other. Heart rate (HR) readings were higher in the ketamine (Group K) group compared to the budesonide (Group B) group, although the difference was not statistically significant. A slight variation in mean arterial pressure (MAP) was observed between the groups at different time intervals. The incidence of sore throat was highest immediately after extubation but decreased over time. At zero hours, two patients in Group B and none in Group K had a sore throat. At two hours, four patients in Group K and five in Group B reported sore throats. At four hours, both groups had three patients with sore throats. At six hours, one patient in Group K and three in Group B reported sore throats. By 12 hours, only one patient in Group B had a sore throat, and none of the patients reported it at 24 hours. The ketamine group had a higher incidence of hallucinations compared to the budesonide group.

Conclusion

Nebulized ketamine and budesonide both effectively reduce POST, with ketamine showing lower severity but higher hallucination rates. Budesonide was associated with fewer complications, making it a preferable option for managing postoperative discomfort.

## Introduction

General anesthesia (GA) is a key aspect of anesthesiology, and endotracheal intubation is one of the most commonly performed procedures under GA to protect the airway from regurgitation and aspiration. A frequent complication of endotracheal intubation under GA is postoperative sore throat (POST) [1]. The incidence of POST in general surgery varies between 12.1% and 70% [2], and it can be as high as 100% in cases involving maxillofacial surgery [3]. POST, an unavoidable side effect of intubation, can range in severity from mild throat irritation to significant pain, difficulty swallowing, and temporary voice changes. Despite being considered a minor issue, patients often regard POST as a major factor negatively impacting their satisfaction with the surgical experience [4-6]. Preventing POST is important as it can adversely affect a patient’s recovery and post-discharge quality of life [5].

POST is typically caused by pharyngolaryngeal mucosa damage during laryngoscopy, nasogastric tube insertion, or oral suctioning [7]. The shape and pressure of the endotracheal tube cuff may also affect tracheal mucosal capillary perfusion, leading to edema and mucosal lesions from contact between the vocal cords and the tracheal tube or the posterior pharyngeal wall [8,9]. Difficult intubation and postoperative nausea and vomiting are additional contributing factors [10].

Pharmacological interventions to prevent POST often involve perioperative medications that reduce inflammation. These include α2-agonists, nonsteroidal anti-inflammatory drugs (NSAIDs), ketamine, corticosteroids, and local anesthetics. Nebulization is favored over other drug delivery routes due to its safety, simplicity, and effectiveness in delivering the medication directly to the lower airways with minimal adverse effects compared to other methods (e.g., intravenous and gargles). Nebulization transforms liquid medication into droplets, with larger particles (10-25 microns) settling in the mouth and throat and smaller ones (5-10 microns) reaching the transition zone between the mouth and airway [11].

Local anesthetics work by reversibly blocking peripheral nerve impulse transmission, while corticosteroids prevent postoperative complications through their anti-inflammatory effects. Among steroids, dexamethasone is the most commonly used in perioperative settings [12]. A meta-analysis by Singh et al., which reviewed 42 trials and compared eight treatment regimens, found that corticosteroid inhalation therapy was one of the most effective in reducing early POST, particularly within four to six hours after extubation [12]. Ketamine has also been shown to reduce the incidence and severity of sore throat [13]. Several studies have investigated the role of ketamine and local anesthetic nebulization in mitigating POST [13]. Thus, the present study aims to assess the effectiveness of preoperative nebulization with ketamine and budesonide in minimizing the incidence of POST at various time intervals.

## Materials and methods

Study design

This hospital-based, prospective, double-blinded, randomized control trial was conducted at Dr. D. Y. Patil Medical College, Hospital and Research Centre, a tertiary-level hospital in Pune, India. Due to a good flow of patients, the study was completed within 12 months (July 1, 2023 to July 1, 2024), including patient enrollment, data collection, and follow-up. Patient enrollment was stopped once the sample size was achieved. The study was approved by the Institutional Ethics Committee of Dr. D. Y. Patil Medical College, Hospital and Research Centre (IESC/PGS/2022/151) and registered with the Clinical Trials Registry of India (CTRI/2023/06/054048). The study adhered to the ethical principles of the Declaration of Helsinki.

Sample size

Using WinPepi version 18, a sample size of 50 patients per group was determined to achieve a power of 80% and a 95% confidence interval for detecting significant differences in the incidence of postoperative sore throat between the two groups. The calculation was based on the study conducted by Devi et al. [14], which observed a 26.7% incidence of sore throat in the lignocaine group compared to 6.7% in the ketamine group at two hours post-extubation.

Participants

Patients between the ages of 18 and 60 years, scheduled for elective surgeries, classified as American Society of Anesthesiologists (ASA) grade I or II, and providing written informed consent were included in the study. Patients who were unwilling to participate, those not meeting the age criteria, or those with preoperative sore throat, upper respiratory tract infection, prior Ryle's tube insertion, known drug allergies, multiple intubation attempts, or any intraoperative or postoperative complications were excluded.

Study procedure

Patients were informed about the study, and written consent was obtained. They fasted for six hours before surgery. The 100 participants were randomized into two groups: Group K (ketamine) and Group B (budesonide). Simple randomization using the lottery method was employed. Nebulization solutions were prepared by an anesthesia assistant who did not participate further in the study.

Intervention, measurements, and data collection

Group K received 50 mg of ketamine (1 ml) plus normal saline (3 ml), and Group B received 250 mcg of budesonide (1 ml) plus normal saline (3 ml) through a nebulization mask, administered over 15 minutes using an oxygen-driven source.

General anesthesia (GA) was induced 15 minutes after nebulization, using IV fentanyl (2 µg/kg) and IV propofol (2 mg/kg), followed by relaxation with IV succinylcholine (2 mg/kg). Laryngoscopy was performed within 15 seconds using a Macintosh blade (size 3 or 4). A sterile, cuffed polyvinyl chloride (PVC) endotracheal tube with an internal diameter of 7-7.5 mm for females and 8-8.5 mm for males was used. Anesthesia was maintained with 33% oxygen in nitrous oxide and sevoflurane. Pain relief was provided with 1 gm IV paracetamol, and IV ondansetron (4 mg) was given 30 minutes before the end of surgery and every eight hours postoperatively. The reversal of neuromuscular blockade was achieved by administering IV neostigmine at a dosage of 0.05 mg/kg, combined with IV glycopyrrolate at a dosage of 0.008 mg/kg. Following this treatment, extubation was performed once the patient regained consciousness.

Outcome measures

Postoperatively, side effects such as hallucinations and POST, were evaluated at zero, two, four, six, 12, and 24 hours after extubation using a four-point scale (0 = no sore throat, 1 = mild, 2 = moderate, 3 = severe). If moderate or severe POST persisted after 24 hours, decongestants and saline gargles were prescribed, with an ENT consultation if symptoms continued. Hemodynamic variables like heart rate (HR) and mean arterial blood pressure (MABP) were recorded at pre-nebulization, post-nebulization, post-induction, and at the same postoperative time intervals.

Statistical analysis

Quantitative data were presented as mean ± standard deviation. The data were analyzed using IBM SPSS Statistics for Windows, Version 20.0 (released 2011, IBM Corp., Armonk, NY). Quantitative data were compared using the unpaired Student's t-test, with a significance level of p < 0.05. Categorical data were analyzed using the chi-square test.

## Results

In the current study, the mean age of the participants was 41.34 ± 12.84 years in Group K and 38.84 ± 13.95 years in Group B, showing no statistically significant difference (t = 0.93, p = 0.353). The mean weight was 74.42 ± 11.44 kg in the ketamine group and 72.36 ± 14.07 kg in the budesonide group (t = 0.80, p = 0.423). There was a male predominance in both groups: 29 males (58%) and 21 females (42%) in Group K and 32 males (64%) and 18 females (36%) in Group B. The groups were comparable in terms of gender distribution (χ² = 0.37, p = 0.269) (Table 1).

**Table 1:** Demographic distribution Age and weight are expressed as mean ± standard deviation. Gender is expressed as qualitative data and analyzed using the chi-square test (χ^2^). The unpaired t-test is used to compare means. A p-value <0.05 was considered statistically significant.

Demographics	Group K	Group B	T-value	P-value
Mean age (Years)	41.34 ± 12.84	38.84 ± 13.95	0.93	0.353*
Weight	74.42 ± 11.44	72.36 ± 14.07	0.80	0.423*
Gender	Group K	Group B	Chi-square value	P-value
Male	29	32	0.37	0.269^#^
Female	21	18		

There was a variation in HRs between the two groups. Group K exhibited higher HRs than Group B at pre-nebulization, post-nebulization, post-induction, and at zero, two, and four hours; however, these differences were not statistically significant. At the sixth and 12th hours, Group B showed higher HRs than Group K, but this was also not statistically significant. At 24 hours, the mean HR was highest in the budesonide group at 77.48 ± 14.55 bpm, compared to 71.62 ± 11.52 bpm in the ketamine group, which was statistically significant (p = 0.027) (Table 2).

**Table 2:** Heart rate across various time points in both groups SD: standard deviation. The unpaired t-test was used for comparison. P < 0.05 = significant.

Time of assessment	Group K (Mean ± SD)	Group B (Mean ± SD)	T-value	P-value
Pre-nebulization	73.84 ± 12.41	72.78 ± 11.47	0.45	0.652
Post-nebulization	75.02 ± 13.48	74.10 ± 11.91	0.38	0.703
Post-induction	77.34 ± 13.91	76.28 ± 14.25	0.021	0.983
0 hour	76.21 ± 13.93	73.15 ± 11.48	1.27	0.206
2 hours	77.65 ± 11.62	75.82 ± 14.91	1.94	0.054
4 hours	74.63 ± 11.50	73.30 ± 11.51	0.60	0.549
6 hours	73.21 ± 12.34	75.50 ± 13.50	0.89	0.372
12 hours	74.28 ± 12.87	75.22 ± 13.59	0.05	0.956
24 hours	71.62 ± 11.52	77.48 ± 14.55	12.23	0.027

A mild variation was observed in the mean arterial pressure (MAP) across all groups at different time intervals. During pre-nebulization, post-nebulization, post-induction, and at zero hours, Group K had a higher MAP than Group B; however, this difference was not statistically significant. At two, four, six, 12, and 24 hours, Group B exhibited higher MAP than Group K, with a statistically significant difference only at six hours, where the t-value was 2.69 and the p-value was 0.008. The remaining readings were not statistically significant. The mean MAP was highest in the ketamine group at 88.71 ± 7.73 during pre-nebulization, while at six hours, it reached 89.28 ± 6.68 in the budesonide group (Table 3).

**Table 3:** Mean arterial pressure across various time points in both groups SD: standard deviation. The unpaired t-test was used for comparison. *Statistically significant (p < 0.05).

Time of assessment	Group K (Mean ± SD)	Group B (Mean ± SD)	T-value	P-value
Pre-nebulization	88.71 ± 7.73	86.26 ± 7.25	1.49	0.137
Post-nebulization	87.82 ± 6.86	86.48 ± 8.70	0.91	0.360
Post-induction	87.54 ± 12.08	87.24 ± 11.24	0.11	0.910
0 hour	86.78 ± 8.60	85.65 ± 9.52	0.622	0.534
2 hours	85.87 ± 7.18	88.93 ± 8.73	1.91	0.058
4 hours	86.29 ± 8.69	87.45 ± 6.91	0.73	0.460
6 hours	85.59 ± 7.01	89.28 ± 6.68	2.69	0.008*
12 hours	86.44 ± 6.80	86.94 ± 6.84	0.366	0.0714
24 hours	86.92 ± 7.75	87.68 ± 6.93	0.843	0.401

**Figure 1:**
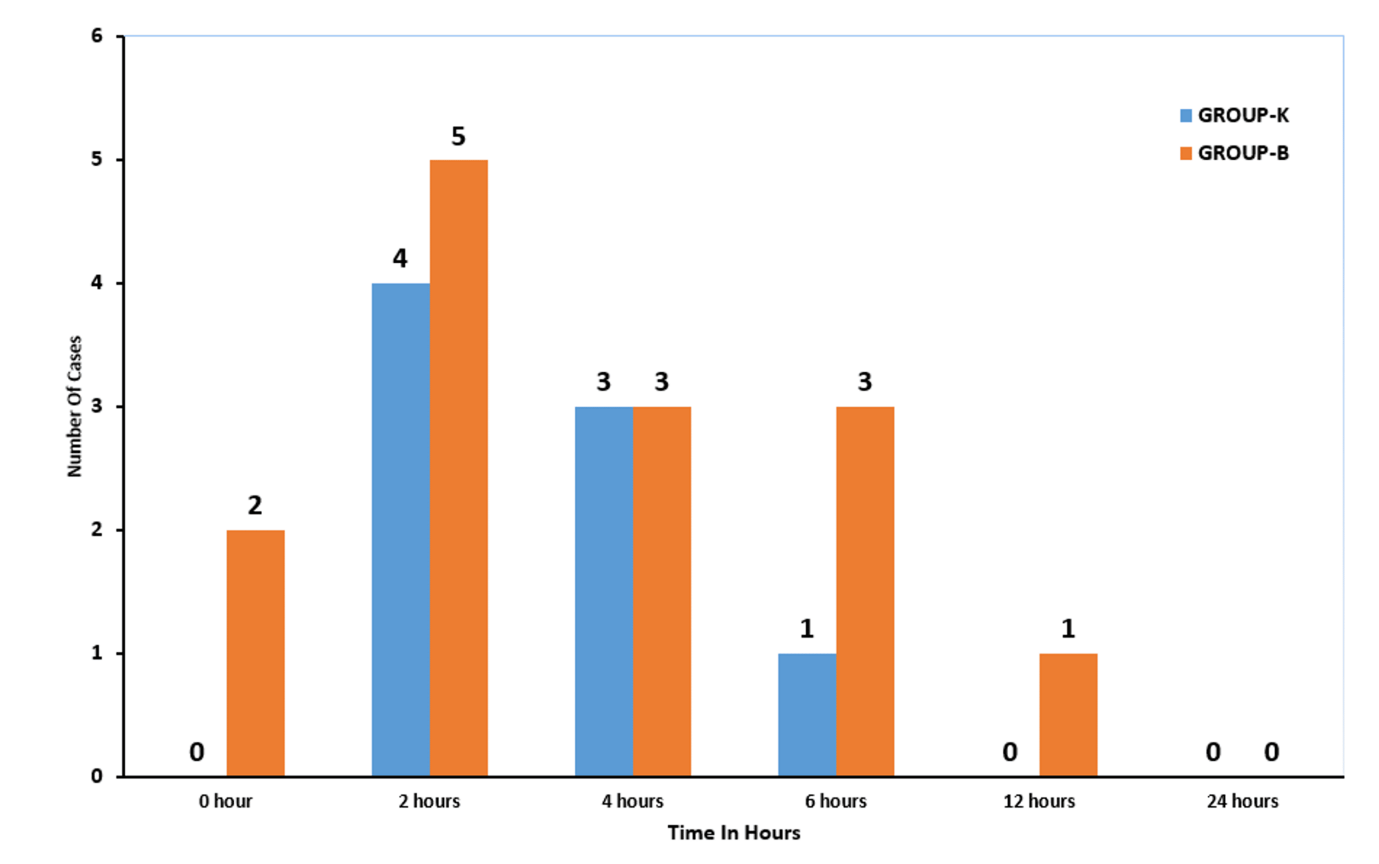
Incidence of sore throat in both groups At 12 hours, Group K had 0 patients; at 24 hours, Group K and Group B had 0 patients.

In our study, sore throat was found highest during the early post-extubation phase and gradually subsided over time. At zero hours, two patients in the budesonide group reported sore throat, while none were reported in the ketamine group. Four patients in Group K and five patients in Group B experienced sore throat at the two-hour mark. Both groups had three patients with sore throat at the four-hour interval. At six hours, one patient in Group K and three patients in Group B reported sore throat. By 12 hours, only one patient in Group B had a sore throat, and by 24 hours, none of the patients in either group reported a sore throat (Figure 1).

**Figure 2:**
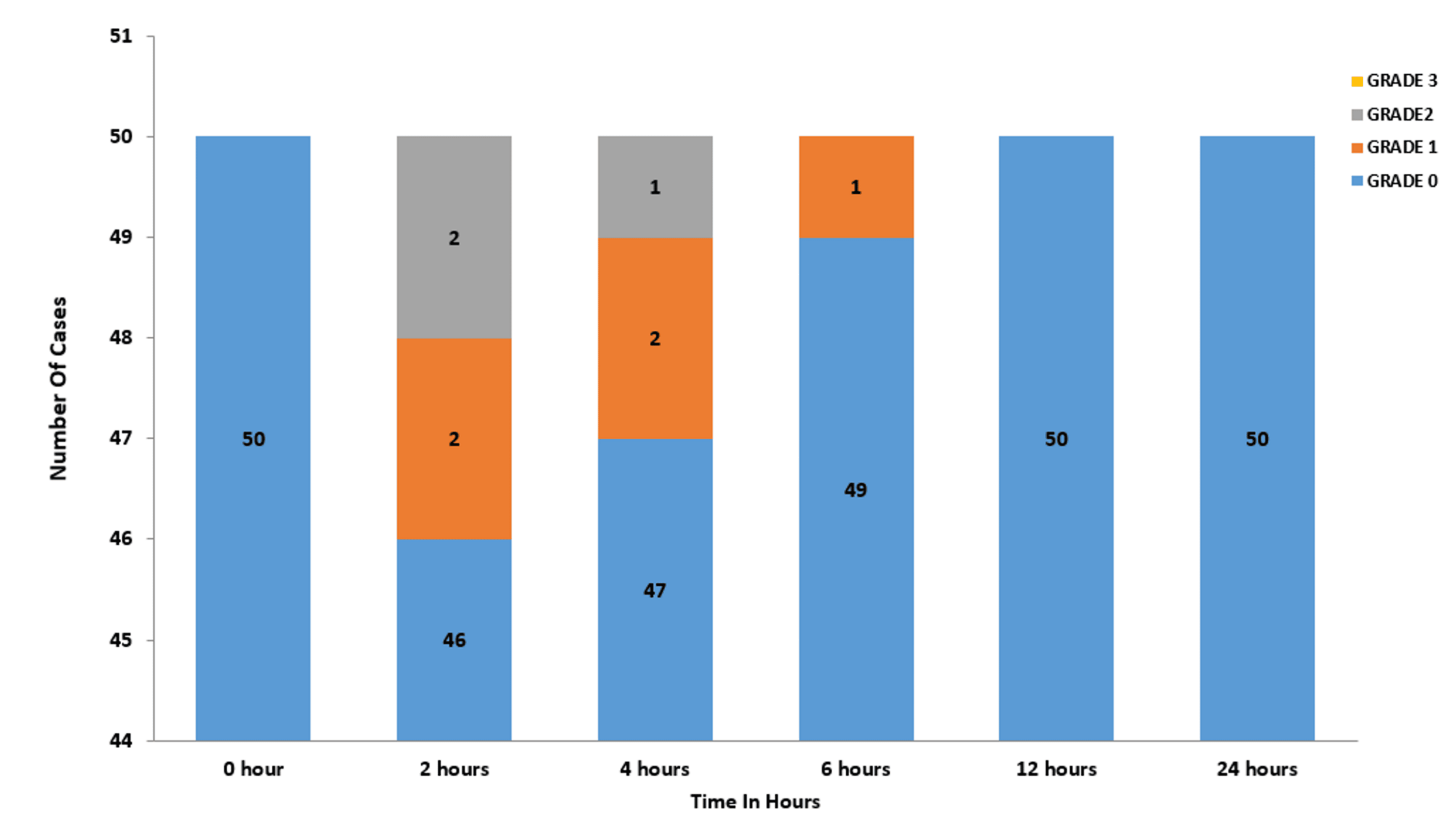
Severity/grading of the sore throat in ketamine group No patients with Grade 3 sore throat in the ketamine group at all time intervals

In the ketamine group, none of the participants reported sore throat at zero hours. At the two-hour mark, 46 participants had grade zero, while two had grade one and two had grade two sore throat. By four hours, 47 participants were at grade zero, two at grade one, and one at grade two. At six hours, 49 participants reported grade zero, with one participant experiencing grade one sore throat. By 12 and 24 hours, all 50 participants in the group had grade zero sore throat (Figure 2).

**Figure 3:**
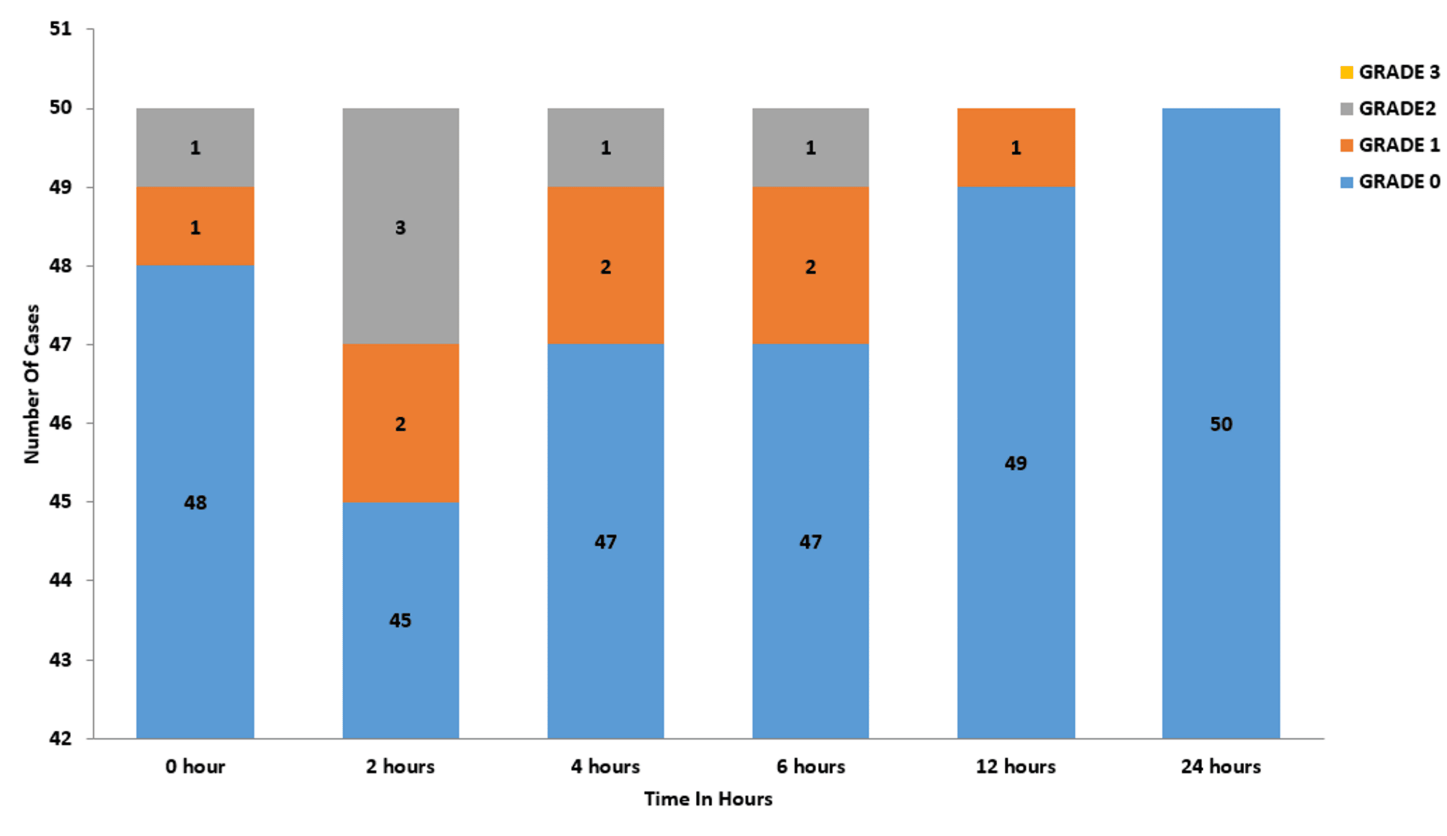
Severity/grading of the sore throat in the budesonide group No patients with Grade 3 sore throat in the budesonide group at all time intervals

In the budesonide group, at zero hours, 48 participants reported grade zero sore throat, while one patient had grade one and another had grade two. At the two-hour mark, 45 participants were at grade zero, two had grade one, and three had grade two sore throat. By four hours, 47 participants were at grade zero, two at grade one, and one at grade two. At six hours, 47 participants again reported grade zero, with two at grade one and one at grade two. At 12 hours, 49 participants had grade zero, with one reporting grade one sore throat. By 24 hours, all 50 participants in the group had grade zero sore throat (Figure 3).

**Figure 4:**
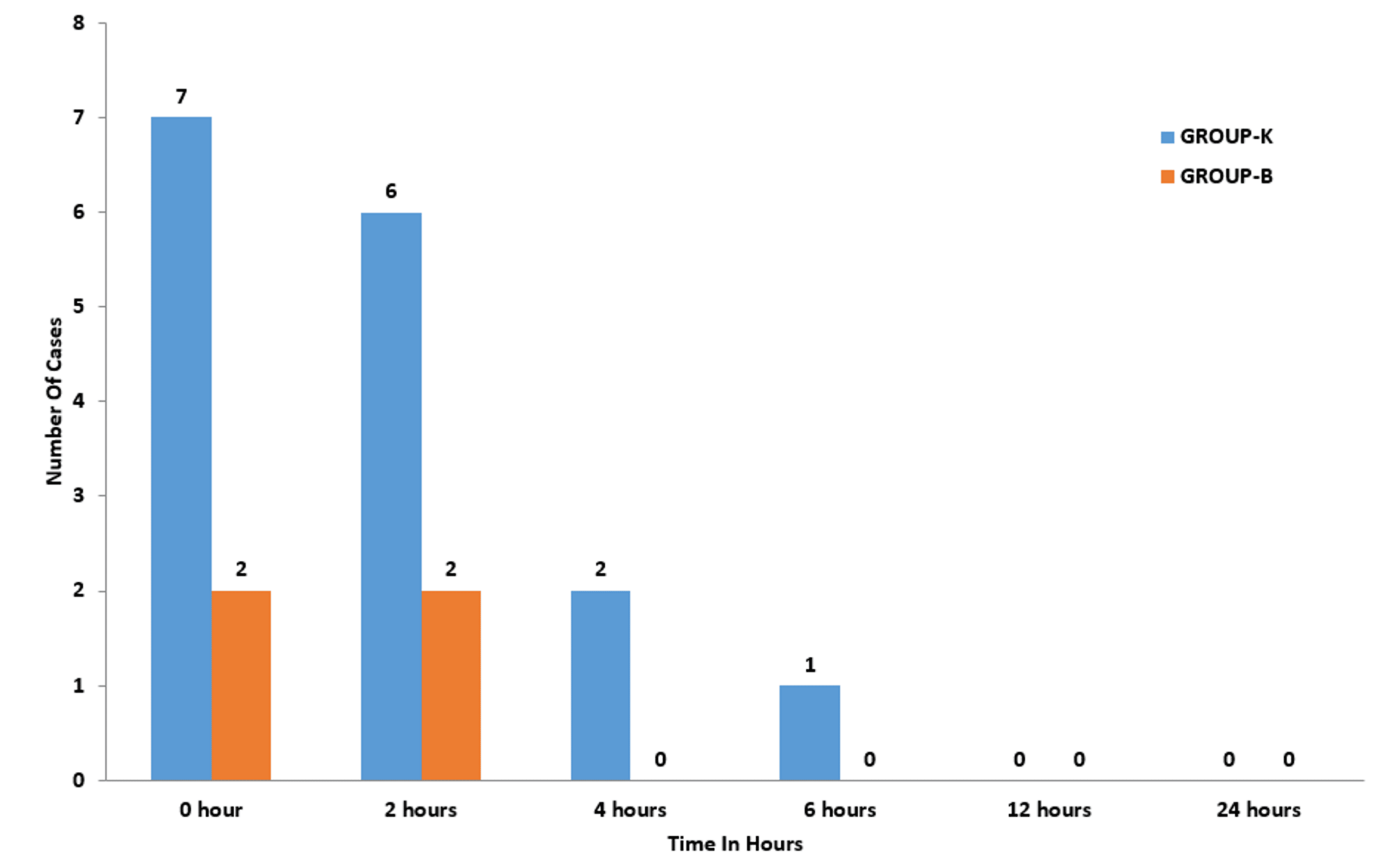
Incidence of hallucinations in both groups At four hours, Group B had 0 patients; at six hours, Group B had 0 patients; at 12 hours, Group K and Group B had 0 patients; at 24 hours, Group K and Group B had 0 patients.

The ketamine group exhibited a higher incidence of hallucinations compared to the budesonide group. At zero hours, seven patients in Group K reported hallucinations, while only two patients in the budesonide group experienced them. At the two-hour mark, six patients in Group K and two in Group B reported hallucinations. From the four-hour mark onwards, no patients in Group B reported hallucinations; however, in Group K, 2 patients had hallucinations at four hours, and one patient reported hallucinations at six hours (Figure 4).

## Discussion

POST is a common complaint following endotracheal intubation, leading to discomfort during the postoperative recovery period. Multiple studies have reported incidence rates ranging from 21% to 66% [15,16]. Factors such as aseptic inflammation, pharyngeal mucosa irritation, and endotracheal tube cuff trauma contribute to the development of POST. Various pharmacological and non-pharmacological interventions have been explored to mitigate this issue [17-19].

In our study, the mean age for Group K was 41.34 ± 12.84 years, while Group B had a mean age of 38.84 ± 13.95 years. By contrast, Mehrotra et al. [20] reported mean ages of 34.3 years for the ketamine group and 36.5 years for the budesonide group. Gender distribution in our study demonstrated a male predominance. Similar findings were reported by Ahuja et al. [21] and Sneha S et al. [22], although Vaghela et al. [23] documented a female predominance in the ketamine group.

In the current study, changes in HR and MAP were comparable between the groups at different time intervals. Initially, ketamine was associated with an increased sympathetic tone, leading to higher HR and MAP than the budesonide group, although these differences were statistically insignificant. At the 24-hour mark, Group B exhibited the highest MAP (87.68), while Group K had an MAP of 86.92. Similar findings were noted by Prasant et al. [24], where the ketamine group reported higher HR and MAP initially. These observations were also supported by Mehrotra et al. [20].

The overall incidence of sore throat in the study population was 22 (22%). The incidence and severity of sore throat at zero hours postoperatively were higher in patients receiving budesonide compared to those receiving ketamine nebulization. Ketamine’s pharmacological properties, including analgesia and sympathetic action, may contribute to its effectiveness. Some studies indicate that ketamine can reduce tumor necrosis factor-alpha production, showcasing its protective mechanism against lung injury by decreasing nitric oxide synthase expression and exhibiting anti-inflammatory properties [24]. Similar findings were reported by Safavi et al. [25]. The N-methyl-D-aspartate (NMDA) receptors, which play a role in nociception and inflammation, are present in the central and peripheral nervous systems and in the spinal cord. The mechanisms and effects of Ketamine may contribute to reducing POST [26].

Numerous studies have examined the incidence of postoperative sore throat. In 2017, Shreesh Mehrotra et al. researched the occurrence of sore throat following nebulization with ketamine and budesonide in patients undergoing GA. They concluded that ketamine was more effective in reducing sore throat in the early postoperative period, while both agents showed improved long-term outcomes. Similarly, our study found a lower incidence and severity of sore throat in the ketamine group at one hour compared to the budesonide group. This effect is likely attributed to ketamine's topical action, which reduces local inflammation, as well as its peripheral effects. Ketamine, as an NMDA receptor antagonist, primarily affects the CNS and parts of the limbic system. Segaran et al. [27] also noted that nebulization with ketamine was more effective than magnesium sulfate in preventing postoperative sore throat. Ketamine’s NMDA receptor antagonism produces anti-inflammatory and antinociceptive effects by acting on NMDA receptors in the pharyngeal wall, an effect not replicated by systemic administration. These findings corroborate our results.

The incidence of postoperative complications, particularly hallucinations, decreased over time. The ketamine group exhibited a higher incidence of hallucinations. Our findings align with those of Sharma et al. [10], who concluded that nausea was more prevalent in the ketamine group. Ketamine can occasionally stimulate the vestibular system, which is responsible for maintaining balance, leading to symptoms like dizziness. Patients often report nystagmus, characterized by rapid eye movement, which is typically observed during dissociative and sub-dissociative states induced by ketamine. In addition, while ketamine primarily impacts NMDA receptors, it also influences dopamine and serotonin receptors, which can contribute to nausea. Alongside its efficacy in reducing POST, another advantage of ketamine is the stability of hemodynamic parameters during endotracheal intubation and extubation.

Strengths of the study

The study highlights the use of nebulization as a drug delivery method, which proves to be beneficial, offering a safe and efficient way to deliver medications directly to the lower airways. It is a non-invasive and simple method to prevent POST. As it is a non-invasive study, patient compliance was good. Nebulization is favored over other drug delivery routes due to its safety, simplicity, and effectiveness in delivering the medication directly to the lower airways with minimal adverse effects compared to other methods. 

Limitations of the study

The sample size of the current study was limited, restricting the ability to draw broader conclusions. In addition, the endotracheal tube (ETT) cuff pressure was not monitored, which is another contributing factor to POST. The study was limited to ASA-I and ASA-II patients, which restricts the applicability of the results to those with higher ASA classifications. In addition, the exclusion of pediatric and elderly patients limits the relevance of the findings to these populations. Another limitation is related to the blinding process. Although efforts were made to blind both participants and healthcare providers, complete blinding may not have been possible, which could introduce bias into the results. The evaluation of postoperative sore throat relied mainly on the patient's subjective reports, lacking objective measurement indicators. Blood concentrations of ketamine and budesonide were not assessed, and the potential effects of systemic drugs on outcomes cannot be dismissed. Further investigation is needed to establish the safety and dosages of the inhaled drugs used. The long-term effects of the medications remain unknown, as patients were only followed up for 24 hours. These limitations should be taken into account when interpreting the results, and further research with a larger sample size and more stringent control of confounding variables is warranted to confirm our findings.

## Conclusions

This study highlights the effectiveness of nebulized ketamine and budesonide in managing postoperative sore throats following endotracheal intubation in elective surgical patients. The findings demonstrate that both treatments have their advantages, with ketamine showing a lower incidence and severity of sore throat compared to budesonide, while budesonide was associated with fewer hallucinations. The results underscore the importance of selecting appropriate pharmacological interventions to enhance patient comfort during the postoperative period. In addition, the use of nebulization as a drug delivery method proves to be beneficial, offering a safe and efficient way to deliver medications directly to the airways.
